# Effect of heat extraction on water‐soluble taste substances in processing products of chilled large yellow croaker (*Pseudosciaena crocea*)

**DOI:** 10.1002/fsn3.1213

**Published:** 2019-11-12

**Authors:** Fen Zhou, Xi‐Chang Wang

**Affiliations:** ^1^ College of Food Science and Technology Shanghai Ocean University Shanghai China

**Keywords:** equivalent umami concentration (EUC), taste activity value (TAV), taste nucleotides, umami amino acids, water‐soluble taste substances

## Abstract

In this paper, we analyzed the umami amino acids composition and content in minced meat (MM), fish head meat (HM), and fish roe (R). According to different ratios of material to liquid, we extracted water‐soluble taste substances from the three materials and then compared composition and content of the umami amino acids and taste nucleotides in the different water‐soluble taste substances. Finally, we analyzed Taste Activity Value (TAV) and the Equivalent Umami Concentration (EUC) between umami amino acids and taste nucleotides. The results showed that umami amino acids total content in MM, HM, and R samples were, respectively, 50.63 mg/100 g, 41.95 mg/100 g, and 67.06 mg/100 g. When the water‐soluble taste substances extracted from the above samples were in the “D” state (MM), the “C” state (HM), and the “C” state (R), respectively, the umami amino acid content could be comparable to the original sample. And the highest EUCs were respectively 1.37 g MSG/100 g, 0.87 g MSG/100 g, and 0.49 g MSG/100 g (MSG: Sodium Glutamate). To some extent, the results of this study indicated that the water‐soluble taste substances could be equivalent to the original sample and could be further applied as a taste regulator in some respects.

## Graphical abstract

The umami amino acid content of the water‐soluble taste substance in the “D” condition extracted from the minced meat was equivalent to the original sample content. The fish head meat and the fish roe were in the “C” state. The “D” condition of MM samples, “C” state of HM samples, and R samples were 1.37 g MSG/100 g, 0.87 g MSG/100 g, and 0.49 g MSG/100 g, respectively, which reached the highest EUCs.

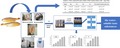

## 1 INTRODUCTION

The breeding of large yellow croaker, *Pseudosciaena crocea* (*P. crocea*), is concentrated in villages along the coast of Zhejiang, Fujian, and Guangdong provinces. With the maturity and improvement of artificial breeding and breeding techniques, the large yellow croaker has become one of the largest marine fish species and the eight major export aquaculture products in China. In 2017, the total production volume of cultured yellow croaker in China reached 17.76 ten thousand tons (Fisheries Administration Bureau, 2018). The large yellow croaker is rich in protein, vitamins, EPA, and DHA and is deeply favored by both domestic and international consumers for its flavor and nutrition (Hui, Liu, Feng, Li, & Gao, 2016; Zheng et al., 2016). However, with the rapid increase in aquaculture production, most of the cultured aquatic products show the trend of decline in quality. Fish qualities are particularly susceptible to the changes of color blurring, loose flesh, and degraded flavor. These problems have seriously affected the healthy development of aquaculture, including market prices and export restrictions. Therefore, researches related to improving fish quality are very important.

In recent years, the aquatic product processing enterprises in China have developed rapidly, the production of by‐products from fish processing has also increased (Liu, Ma, Wang & Qin, 2017). A variety of biologically active substances, such as collagen, chitin, gelatin, lipase, protease, and biologically active peptides exit in the fish processing by‐products (Saeleaw & Benjakul, 2017; Jridi et al., 2018; Balti et al., 2015; Sila et al., 2015; Chi et al., 2015). Therefore, it is possible to make full use of the active substances in these processing by‐products, which could improve the utilization rate and simultaneously reduce the environmental pollution.

Taste is one of the important indicators for evaluating the quality of food. Taste substances are generally hydrophilic and consist of small molecules/ions. The main water‐soluble taste substances that contain amino acids, small peptides, nucleotide metabolites, inorganic salts, and sugars are responsible for the taste of meat and meat products (Zhang, Wang, Liu, Xu, & Zhou, 2012). Umami has a unique savory taste and is often used as an indicator of meat quality (Yasumatsu et al., 2015; Zhang, Pan, Venkitasamy, Ma, & Li, 2015). Substances similar in structure elicit similar tastes, it is extracted from foods into the saliva, which then interact with specific taste receptors/channels for each basic taste (Kawai, Uneyama, & Miyano, 2009). In addition, the key compound that produces umami is inosine monophosphate (IMP) and its degradation products (ribose and hypoxanthine)(Bagnasco et al., 2014; Kavitha & Modi, 2007; Vani, Modi, Kavitha, Sachindra, & Mahendrakar, 2006). Glutamate (Glu) is a typical umami compound, and other substances such as free amino acids and flavor peptides can also elicit umami (Yu, Zhang, Miao, Li, & Liu, 2016). The special taste characteristics of the water‐soluble substances can improve the palatability of food; thus, it could be further developed into a safe substitute for high sodium seasoning (Zhu et al., 2008).

In the previous experiments, we selected minced meat, fish head meat, and fish roe from seven by‐products (minced meat, fish head meat, fish roe, viscera, fish ventral skin, fish dorsal skin and fish scale)according to the content and composition of taste amino acid and nucleotide. Therefore, this experiment extracted the taste substance from the minced meat, fish head meat, and fish roe samples by heat treatment. The taste substance is mainly water‐soluble taste compound. This experiment used amino acid analyzer and combined with high‐performance liquid chromatography (HPLC) equipment to analyze the content of umami amino acids in water‐soluble taste compound. The purpose of this paper is to evaluate whether the extracted water‐soluble taste substances can be used as substitute original sample as taste substance to be added to other substances.

## 2 MATERIALS AND METHODS

### 2.1 Materials and sample preparation

**Figure 1:**
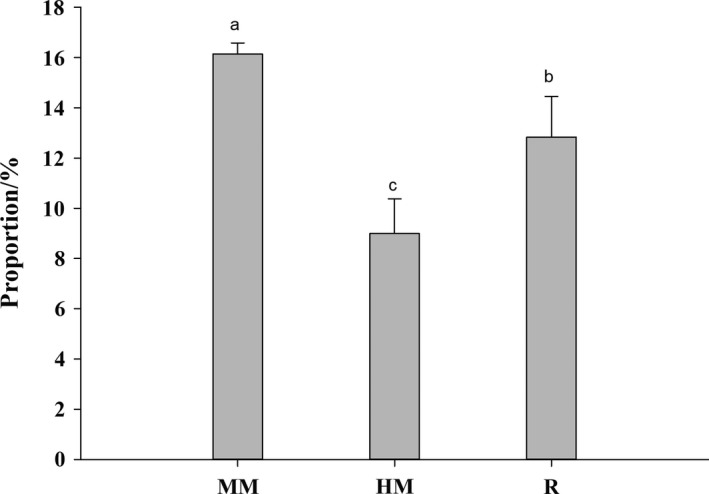
Account for the proportion of the entire fish. Minced meat—MM, Fish head meat—HM, Fish roe—R. Results with different letters (a–c) were significantly different in by‐products at *p < *.05 by Duncan's multiple range test

Fresh fish (*P. crocea*) (*n* = 40; 374.82 ± 11.83 g; 31.43 ± 0.86 cm) was obtained from the Qimin agriculture and industry co. LTD, Fujian Province (China). When fish was obtained, it was firstly washed using the flowing water. The minced meat (MM) was taken from the skin and fish tail. The minced meat, fish head meat (HM), and fish roe (R) were stored in a sealed plastic bag at −40°C until using. The minced meat, fish head meat, and fish roe were used as the research subject. The samples account for the proportion of whole fish were summarized in Figure 1. Before test, the research subject was cut into small pieces after thawing in a refrigerator (4°C).

Trichloroacetic acid (TCA), Sodium hydroxide (NaOH), Perchloric acid (PCA), Potassium hydroxide (KOH), 5'‐adenosine monophosphate (5′‐AMP), and 5'‐inosine monophosphate (5′‐IMP). All chemicals were used in the measurement. All solutions were prepared by ultrapure water.

### 2.2 The biological measurement and chemical analyses

The MM, HM, and R samples were analyzed for proximate composition following Chinese standard methods. Fat and moisture contents were analyzed by GB/T 1477.2‐1993 (Meat and meat products—Determination of free fat content) and GB/T 5009.3‐2010 (Meat and meat products Determination of moisture content), respectively. Total protein was determined by the Kjeldahl method using a nitrogen conversion factor of 6.25 (Robert, 2006; Yaich et al., 2011). Ash was determined by GB/T 5009.4‐2016 (Meat and meat products Determination of moisture content). The determination of condition factor is based on the following formula.

$$\text{Condition}\;\text{factor}:\;K\left(\%\right)\;=\;\left(W/L^{3}\right)\;\times \;100,$$

where *W* is the body weight (g) and *L* is the body length (cm).

### 2.3 The water‐soluble taste substances preparation

The water‐soluble taste substances were extracted, respectively, from the MM, HM, and R samples well prepared before. Firstly, the samples and the ultrapure water were mixed according to the different ratio of the sample to the liquid and homogenized for 30 s. Then, the mixed samples were ultrasound 10 min and kept at 100°C water bath for 1 hr to extract the water‐soluble taste substance. Lastly, the mixtures above were filtered through a Whatman.No.54 filter membrane and the filtrate was cooled and centrifuged at 10614 g for 20 min at 4°C. And then, the supernatants obtained from the centrifuged filtrate were water‐soluble taste substance for analyzing. Stressing that in order to achieve umami amino acids content as similar as in the original sample, we concentrate the taste substance. The specific concentration treatment were as follows: firstly, the water‐soluble taste substance was extracted under the ratio of material to liquid 1:4 (g/ml); “A” was the supernatant extracted under the ratio of material to liquid 1:2 (g/ml); “B” was the water‐soluble taste substance extracted by using A as the extract liquid under the ratio of material to liquid 1:2 (g/ml); “C” was the water‐soluble taste substances extracted by using B as the extract liquid under the ratio of material to liquid 1:2 (g/ml); “D” was the water‐soluble taste substance extracted by using C as the extract liquid under the ratio of material to liquid 1:2 (g/ml); and “T” was unextracted original sample as the control treatment.

### 2.4 The free amino acid analysis

The composition and concentration of free amino acid (FAA) in all samples were determined according to Chen, Zhang, and Shrestha (2007). Seventeen amino acid standard samples were purchased from Sigma (Sigma Chemical Co., St. Louis, MO, USA). For each sample, 2.00 g (2.00 ± 0.01 g) was weighed into an 50 ml centrifuge tube and homogenized with 15 ml of 5% TCA. Samples should be allowed to stand for 2 hr, after ultrasound for 5 min, and supernatant was collected by centrifugation (4°C, 10,000 r/min, 10 min). 5 ml of supernatant was removed to adjust pH to 2.0 with 6 mol/L NaOH and 1 mol/L NaOH. These supernatants were filled with ultrapure water to 10 ml, and 1 ml was filtered through a membrane filter (0.2 μm) for quantitative analysis using an amino acid analyzer (L‐8800, Hitachi, Japan). The analytical conditions were as follows: chromatographic column size (4.6 × 150 mm, 7 μm); column temperature: 50°C; 1 channel flow rate: 0.4 ml/min; and 2 channel flow rate: 0.35 ml/min. The umami amino acids were used as our analysis result.

### 2.5 The taste nucleotides analysis

The nucleotide analysis was carried out using the same UPLC system with the organic acid analysis (LC‐2010CHT, Shanghai specimen factory, China). Samples were prepared using methods followed of Yokoyama, Sakaguchi, Kawai, and Kanamori (1992) with minor adjustments. During pretreatment, 10 ml of PCA was added to 5.00 g sample in 50 ml centrifuge tube, homogenized and then ultrasound for 5 min. The supernatant was saved after centrifuged at 10614 g for 3 min at 4°C. The treatment of sedimentation was repeated as the above steps. The two times supernatants were combined, and the pH of the supernatants were adjusted to 5.8 with 6 mol/L KOH. After placing 30 min, the final volume was brought up to 50 ml with ultrapure water and filtered through a membrane filter (0.2 μm) for quantitative analysis. The analytical conditions were as follows: chromatographic column size (4.6 × 250 mm, 5 μm; ODS‐3); the mobile phase was methanol‐Buffer salt II (5:95, v:v) with a flow rate of 1.0 ml/min; column temperature: 28°C; sample intake: 10 μl; and detection wavelength: 254 nm.

### 2.6 The Umami taste evaluation method

Taste Activity Value (TAV) was calculated using the following equation: TAV = C/T.

C: absolute concentration of umami taste substance, mg/100 g; T: umami threshold of umami taste substance, mg/100 g (5′‐IMP = 25 mg/100 g, 5′‐AMP = 50 mg/100 g)( Chen & Zhang, 2007).

The equivalent umami concentration (EUC, g MSG/100 g) is the concentration of MSG equivalent to the umami intensity given by a mixture of MSG‐like amino acids and the 5′‐nucleotide and is represented by the following formula. (Chen et al., 2007).

$$\text{EUC}\;=\;\sum \text{aibi}\;+\;1218\left(\sum \text{aibi}\right)\left(\sum \text{ajbj}\right),$$

where ai is the concentration (g/100 g) of each umami amino acid (Asp, Glu); aj is the concentration (g/100 g) of each umami 5′‐nucleotide (5′‐IMP, 5′‐AMP); bi is the relative umami concentration (RUC) of each umami amino acid relative to that of MSG (Asp, 0.077 and Glu, 1); bj is the RUC of each umami 5′‐nucleotide relative to that of MSG (5′‐IMP, 1 and 5′‐AMP, 0.18); and 1218 is a synergistic constant based on the concentration (g/100 g) used.

### 2.7 Statistical analysis

Three replicates were done for each experiment. Results were presented as mean ± standard deviations. Data were subjected to analysis of variance (ANOVA) using SPSS 13.0 software. All the figures in the text used Sigma Plot12.5 software.

## 3 RESULTS AND DISCUSSION

### 3.1 Analysis of the chemical composition

The condition factor or fatness could reflect the growth and development of fish. In this study, we observed the condition factor of the large yellow croaker could reach 1.85%. This indicated the yellow croakers grew better. Among the processed products of the yellow croakers, the proportion of the MM, HM, and R were larger in the whole fish, relatively. In addition, the account proportion of MM, HM, and R in the whole group of large yellow croakers showed MM (16.48%)>R (11.95%)>HM (8.38%), and there were significant differences among the three samples (*p* < .05).

Protein, moisture, and fat are important functions and nutritional components of fish and fish products. The proximate composition of the MM, HM, and R samples were analyzed and averaged to obtain a reference value, relatively. Table 1 showed the average of different sample sets. The moisture contents in HM samples (78.30%) were significantly higher than the R and MM samples. The crude protein varied in a closer range in R samples (13.28%) and MM samples (13.50%). Fish roe are natural food raw materials with characteristics of typical high protein and low fat. And, the crude fat content in R samples was similar to Siberian sturgeon (14%~20%) studied by GisbertWilliot and Castelló‐Orvay (2010). The crude protein content in R samples (25.72%) was also similar to Siberian sturgeon (20.38%~26.00%) studied by Gisbert et al. (2010). Caviar processed from Siberian sturgeon is a well‐known high‐end Chinese and foreign nutritious food.

**Table 1:** Analysis of the basic nutrient components of different by‐products of chilled *Pseudosciaena crocea* (%, $\bar{x}$ ± s, *n* = 3)

Sample	Moisture	Crude fat	Crude protein	Ash
MM	69.64 ± 0.34^b^	13.50 ± 0.68^a^	18.07 ± 0.30^b^	1.12 ± 0.02^b^
HM	78.30 ± 0.07^a^	5.38 ± 0.31^b^	14.40 ± 0.67^c^	0.91 ± 0.07^b^
R	55.84 ± 0.02^c^	13.28 ± 0.65^a^	25.72 ± 0.14^a^	2.23 ± 0.30^a^

Note

Different letters (a–c) in the same column indicate significant differences (*p* < .05), *n* = 3.

Abbreviations: HM, Fish head meat; MM, Minced meat; R, Fish roe.

### 3.2 Analysis of the free amino acids composition and content

Amino acids play important role in the growth and development of organisms, they can enhance the taste characteristics of foods and stimulate the taste of consumers. Amino acids also can participate in the development of taste indirectly and could influence the nutritive values and taste in the muscle of fish (Hang et al., 2016; Kong et al., 2017; Jiang et al., 2016; Bermúdez et al., 2014). The free amino acids served as significant contributors to taste and important precursors for aroma compound and influence osmotic regulation and buffering capacity in the cells of marine animal (Harimana et al., 2018). Free amino acids and short‐chain peptides have nutraceutical effects and can promote maintenance of amino acids profiles (Chalamaiah, Hemalatha, & Jyothirmayi, 2012).

Among amino acids, Aspartate (Asp) and Glutamic acid (Glu) are umami amino acids (Liu, Liu, He, Song, & Chen, 2015). Besides, Glycine (Gly) and Alanine (Ala) are also thought to contribute to the umami taste of seafood providing some sweetness (Gong et al., 2016). Thus, the umami amino acid content we mentioned is the total content of the four amino acids. Table 2 showed the free amino acid content in the MM, HM, and R samples before the heating extracting. If the amino acid content is lower than the taste threshold value, it only shows the effect of enhancing the flavor of food, but when the content is higher than the taste threshold, the umami can be produced. From Table 2, the total content of umami amino acid in R samples could reach 67.06 mg/100 g, it was higher compared with the MM samples (50.63 mg/100 g)and HM sample (41.95 mg/100 g). The Glu content in R samples was significantly higher than others (*p* < .05). Glutamic acids containing di‐ and tri‐peptides have been of particular interest because they are related to the unique taste of umami (Dang, Gao, Ma, & Wu, 2015). However, the Gly content of the fish roe was significantly lower than the MM and HM samples (*p* < .05). The taste threshold of Glu is 30 mg/100 g and the TAV values of Glu in R samples were greater than 1(1.16), the compounds whose TAV was greater than 1 were considered as active in food taste, the larger the value, the more significant the influence (Kong et al., 2017; Zhao, Jiang, Xu, & Xia, 2017). Therefore, Glu has a significant contribution to the umami taste characteristics of the R samples. Besides, the total content of free amino acids and essential amino acids in R samples were, respectively, significantly greater than that of MM and HM samples. Glu is an important umami amino acid in brain tissue biochemical metabolism and participates in the production of a variety of physiologically active substances (Borroto‐Escuela, Tarakanov, Brito, & Fuxe, 2018).

**Table 2:** Analysis of the composition and content of free amino acids in the original sample (mg/100 g, $\bar{x}$ ± s, *n* = 3)

Amino acid	MM	HM	R
Aspartate (Asp)	2.15 ± 0.50^b^	2.00 ± 0.33^b^	7.06 ± 0.49^a^
Threonine (Thr)*	7.43 ± 1.76^b^	8.03 ± 1.26^b^	11.53 ± 0.81^a^
Serine (Ser)	7.16 ± 1.65^b^	7.03 ± 1.14^b^	10.56 ± 0.71^a^
Glutamic acid (Glu)	12.48 ± 2.80^b^	12.03 ± 2.00^b^	34.99 ± 2.25^a^
Glycine (Gly)	21.64 ± 4.91^a^	17.13 ± 2.93^a^	7.98 ± 0.49^b^
Alanine (Ala)	14.36 ± 3.16^ab^	10.79 ± 1.95^b^	17.02 ± 1.61^a^
Cysteine (Cys)	0.58 ± 0.17^b^	0.54 ± 0.12^b^	1.38 ± 0.07^a^
Valine (Val)*	2.41 ± 0.56^b^	2.67 ± 0.49^b^	11.09 ± 0.81^a^
Methionine (Met)*	1.01 ± 0.25^b^	1.12 ± 0.21^b^	5.54 ± 0.42^a^
Isoleucine (Ile)*	1.62 ± 0.28^b^	1.86 ± 0.34^b^	7.41 ± 0.54^a^
Leucine (Leu)*	2.48 ± 0.43^b^	2.59 ± 0.46^b^	18.80 ± 1.41^a^
Tyrosine (Tyr)	1.56 ± 0.04^b^	1.61 ± 0.26^b^	7.55 ± 0.47^a^
Phenylalanine (Phe)*	1.91 ± 0.06^b^	2.01 ± 0.22^b^	11.04 ± 0.84^a^
Lysine (Lys)*	13.02 ± 2.90^a^	13.29 ± 1.59^a^	12.86 ± 0.86^a^
Histidine (His)	3.51 ± 0.84^c^	10.03 ± 1.57^b^	16.62 ± 1.00^a^
Arginine (Arg)	0.69 ± 0.14^b^	0.90 ± 0.02^b^	15.35 ± 0.74^a^
Proline (Pro)	7.83 ± 1.80^ab^	9.54 ± 1.31^a^	8.73 ± 0.55^a^
UFA	50.63 ± 11.37^ab^	41.95 ± 7.20^b^	67.06 ± 4.82^a^
EAA	29.88 ± 6.12^b^	31.57 ± 4.31^b^	78.27 ± 5.68^a^
FAA	101.84 ± 62.24^b^	103.16 ± 6.99^b^	205.51 ± 4.01^a^

Note

Different letters (a,b) in the same column indicate significant differences (*p* < .05), *n* = 3.

Abbreviations: EFA, Essential free amino acid; FAA, Total amount of free amino acids; HM, Fish head meat; MM, Minced meat; R, Fish roe; UFA, Umami Free Amino acids.

**Figure 2:**
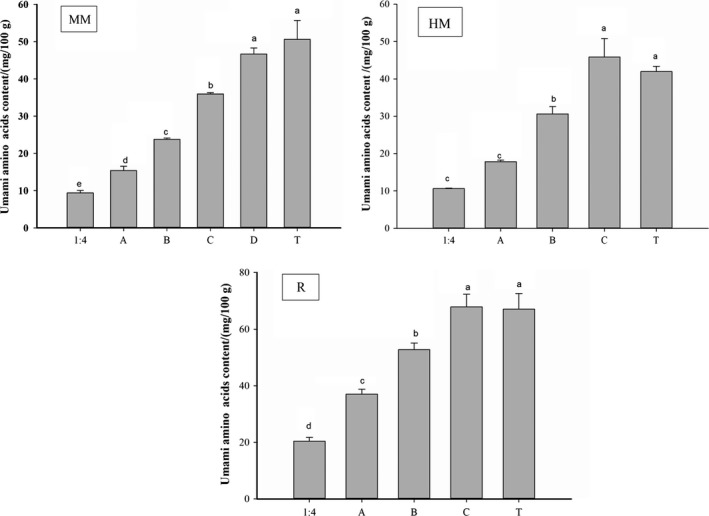
Total content of umami amino acids in water‐soluble taste substance. Different letters (a–e) in the same column indicate significant differences (*p* < .05), *n* = 3. “MM”: Minced meat; “HM”: Fish head meat; and “R”: Fish roe; “1:4”: Under feed liquid ratio1:4; “A”: Under feed liquid ratio1:2; “B”: Under feed liquid ratio1:2, using A as the extract liquid; “C”: Under feed liquid ratio1:2, using B as the extract liquid; “D”: Under feed liquid ratio1:2, using C as the extract liquid; and “T”: Unextracted original sample

Figure 2 showed the total content of umami amino acids in water‐soluble taste substance from the minced meat, fish head meat, and fish roe by heating extracting, respectively. In the process of heat extraction, the degeneration of myofibrillar proteins in samples lead to the accumulation and shortening of myofibrils and lead to the continuous dissolution of soluble proteins such as myosin, sarcoplasmic proteins, and actomyosin. The protein would degrade and produce amino acids, peptides, and other taste substances (Tang, Li, Li, Zhang, & Chen, 2015). From Figure 2, the content of umami amino acids gradually increased with repeated heat extraction in all samples. As the HM samples reach to “C” state, the total content of umami amino acids was 3.90 mg/100 g higher than that of fish head meat (“T”, 41.95 mg/100 g). The R samples reached to “C” state, it was 4.53 mg/100 g higher than that of fish roe (“T”, 67.06 mg/100 g). However, water‐soluble taste substance extracted by the minced meat reached the “C” state, the total content of the umami amino acid with umami characteristics was still much lower than that of the original sample (“T”, 50.63 mg/100 g), it was 3.93 mg/100 g lower than the minced meat original sample. Therefore, we continued to heat extract and concentrate to the “D” state. At this time, the content was still significantly lower than the original sample, but the Gly content in the “D” state was significantly lower (*p* < .05) than that in the “C” state (Table 3). Thus, we finally adopt water‐soluble taste substances obtained in the “D” state. In addition, the “C” state in HM and R sample and the “D” in MM sample, the total content of umami amino acids all were not significantly different from that in the original sample, respectively. It was indicated that the umami amino acid content in the water‐soluble taste substance could be equivalent to that in the original sample under this heat extraction condition. We can use water‐soluble taste substance in this state to replace the fish head meat and the fish roes to achieve the umami taste.

**Table 3:** Composition and content analysis of umami amino acids in water‐soluble taste substance (mg/100 g, $\bar{x}$ ± s, *n* = 3)

Sample	Proportion	Asp	Glu	Gly	Ala
MM	1:4	0.89 ± 0.07^d^	2.80 ± 0.22^e^	2.85 ± 0.23^e^	2.83 ± 0.13^e^
	A	1.65 ± 0.10^c^	4.76 ± 0.31^d^	4.81 ± 0.27^d^	4.20 ± 0.28^d^
	B	2.67 ± 0.07^b^	7.37 ± 0.12^c^	6.98 ± 0.18^c^	6.75 ± 0.05^c^
	C	2.83 ± 0.25^b^	8.45 ± 0.34^b^	16.28 ± 0.55^a^	8.38 ± 0.54^b^
	D	6.47 ± 0.19^a^	14.15 ± 0.36^a^	12.24 ± 0.29^b^	13.5 ± 0.38^a^
HM	1:4	2.41 ± 0.02^c^	2.31 ± 0.02^d^	3.49 ± 0.01^d^	2.43 ± 0.10^d^
	A	3.98 ± 0.07^bc^	3.90 ± 0.10^c^	5.94 ± 0.10^c^	4.02 ± 0.07^c^
	B	6.83 ± 0.22^b^	6.57 ± 0.43^b^	9.74 ± 0.62^b^	7.48 ± 0.55^b^
	C	11.9 ± 0.46^a^	9.58 ± 0.43^a^	13.67 ± 1.15^a^	10.66 ± 1.09^a^
R	1:4	2.35 ± 0.14^d^	10.30 ± 0.67^d^	2.17 ± 0.10^d^	5.57 ± 0.25^d^
	A	4.47 ± 0.18^c^	18.72 ± 0.86^c^	3.86 ± 0.14^c^	9.99 ± 0.34^c^
	B	6.35 ± 0.24^b^	27.10 ± 1.23^b^	5.50 ± 0.16^b^	13.82 ± 0.40^b^
	C	7.77 ± 0.45^a^	39.08 ± 3.51^a^	6.79 ± 0.42^a^	17.95 ± 0.41^a^

Note

Different letters (a–e) in the same column indicate significant differences (*p* < .05), *n* = 3.

“1:4” was the supernatants under feed liquid ratio1: 4 (g/ml); “A” was the supernatants under feed liquid ratio1: 2 (g/ml); “B” was water‐soluble taste substances using A as the extract liquid under feed liquid ratio1: 2 (g/ml); “C” was water‐soluble taste substances using B as the extract liquid under feed liquid ratio1: 2 (g/ml); and “D” was water‐soluble taste substances using C as the extract liquid under feed liquid ratio1: 2 (g/ml).

Abbreviations: HM, Fish head meat; MM, Minced meat; R, Fish roe.

The Table 3 showed the composition and content of umami amino acids in water‐soluble taste substances during different heat extractions. In MM samples, the content of Asp, Glu, Gly and, Ala gradually increased with repeated heat extraction in all samples, except for the Gly content. The water‐soluble taste substance in the minced meat was extracted from the “C” state to the “D” state, and the content of Gly was significantly decreased. The Asp, Glu, and Ala content in the “D” state in the MM samples were higher than the original sample. And, the umami amino acids (Asp, Glu, Gly and Ala) in the “C” state in the R samples were all higher than the original sample. However, in HM samples, the Asp content in “1:4”, “A” state, “B” state, and “C” state were higher than the original sample.

### 3.3 Analysis of the taste nucleotides content and EUC

Nucleotides are important low molecular compounds with many special physiological functions in living organisms. Purine nucleotides, such as guanine monophosphate (GMP), inosine monophosphate (IMP), and adenosine monophosphate (AMP) can enhance the umami taste significantly (Liu, Xu, & Zhou, 2007; Yamaguchi & Ninomiya, 2000). In the three taste nucleotides, the content of 5'‐IMP is significantly higher than that of 5'‐AMP and 5'‐GMP, because 5'‐IMP is mainly found in animal foods, and 5'‐GMP is mainly umami substances found in plant foods, such as fungi.

Adenosine triphosphate has an advantage in live fish muscles, after death, it soon degrades into related associations such as ADP, AMP, and IMP and these associations affect the taste of fish. IMP and AMP are the two main taste nucleotides in fish (Qiu, Chen, Xie, Qu, & Song, 2016), both of which can be produced by the decomposition of adenosine triphosphate (ATP) (Sun, Zhang, Li, Li, & Xie, 2013). Among them, IMP is an extremely umami flavoring agent in meats such as fish meat. As an umami substance, IMP is involved in the phosphorylation of the 5'‐position in its molecular structure. 5'‐IMP has synergistic effect with MSG, and the combination of the two has obvious strong umami effect (Mohan, Ravishankar, Tksrinivasa, & Kashok, 2009). For example, 5'‐IMP and MSG can be mixed at a ratio of 1:5 (g/g) to 1:20 (g/g), the umami taste of sodium glutamate increased by 6 times.

During heat processing, the umami amino acid content in the water‐soluble taste substances could reach the original sample, the composition and content of the taste nucleotide are shown in Table 4 The content of IMP gradually increased with repeated heat extraction in all samples significantly (*p* < .05). However, the content of AMP did not change significantly during the whole heat extraction process. To evaluate these compounds for their taste impact, TAV was a very useful index. By mixing the minced meat with ultrapure water at the ratio of 1:2 (“A” condition), the content of IMP was greater than its threshold (25 mg/100 g) (Kato, Rhue, & Nishimura, 1989). And, when the HM sample reached the “B” state, the content of IMP was also greater than its threshold (25 mg/100 g). In the MM sample, from the “A” state to the “D” state, whose TAVs were ranged from 1.25 to 3.01. And, the HM sample, the TAVs in the “B” state and “C” state were 1.16 and 2.66, respectively. The content of IMP in the R samples were low, the TAV value were less than 1. In addition, the TAV value of AMP in all samples were also less than 1. Therefore, in the case of taste nucleotides, MM and HM were concentrated to obtain the water‐soluble taste substance, mainly IMP. With decreasing of the IMP concentration, the taste of the fish gradually becomes unacceptable, and as the IMP degrades, the resulting HX produces an unacceptable bitter taste. When the umami amino acid content in the water‐soluble substance can be comparable to the original sample, that is, the MM sample was in the “D” state, the HM and R sample were in the “C” state, the content of HX in the all sample were higher than in other condition (Data not shown).

**Table 4:** Composition and content analysis of taste nucleotides in water‐soluble taste substance (mg/100 g, $\bar{x}$ ± s, *n* = 3)

Sample	Proportion	IMP (mg/100 g)	Thresholds (mg/100 g)	TAV	AMP (mg/100 g)	Thresholds (mg/100 g)	TAV
MM	1:4	16.88 ± 0.34^d^	25.00	0.68	4.71 ± 0.03^c^	50.00	0.09
	A	31.17 ± 0.17^c^	25.00	1.25	6.03 ± 0.04^a^	50.00	0.12
	B	42.55 ± 0.11^b^	25.00	1.70	5.24 ± 0.78^bc^	50.00	0.10
	C	43.68 ± 1.03^b^	25.00	1.75	5.38 ± 0.03^abc^	50.00	0.11
	D	75.23 ± 2.63^a^	25.00	3.01	5.77 ± 0.12^ab^	50.00	0.12
HM	1:4	14.08 ± 0.10^d^	25.00	0.56	4.67 ± 0.06^c^	50.00	0.09
	A	22.64 ± 0.72^c^	25.00	0.91	6.15 ± 0.10^a^	50.00	0.12
	B	29.12 ± 0.24^b^	25.00	1.16	6.11 ± 0.08^a^	50.00	0.12
	C	66.44 ± 3.25^a^	25.00	2.66	5.74 ± 0.19^b^	50.00	0.11
R	1:4	4.35 ± 0.13^c^	25.00	0.17	4.17 ± 0.10^b^	50.00	0.08
	A	4.75 ± 0.15^c^	25.00	0.19	4.64 ± 0.02^b^	50.00	0.09
	B	5.71 ± 0.50^b^	25.00	0.23	5.39 ± 0.25^b^	50.00	0.11
	C	7.02 ± 0.13^a^	25.00	0.28	12.57 ± 1.61^a^	50.00	0.25

Note

Different letters (a–d) in the same column indicate significant differences (*p* < .05), *n* = 3.

“1:4” was the supernatants under feed liquid ratio1: 4 (g/ml); “A” was the supernatants under feed liquid ratio1: 2 (g/ml); “B” was water‐soluble taste substances using A as the extract liquid under feed liquid ratio1: 2 (g/ml); “C” was water‐soluble taste substances using B as the extract liquid under feed liquid ratio1: 2 (g/ml); and “D” was water‐soluble taste substances using C as the extract liquid under feed liquid ratio1: 2 (g/ml).

Abbreviations: HM, Fish head meat; MM, Minced meat; R, Fish roe.

**Figure 3:**
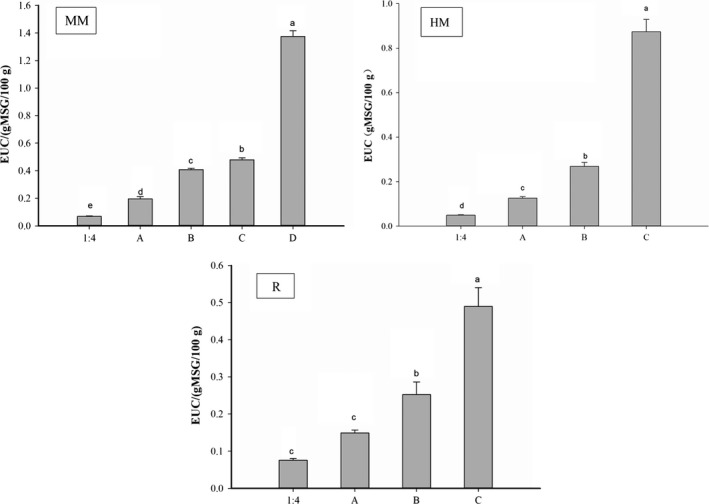
The EUC values in water‐soluble taste substance. Different letters (a–e) in the same column indicate significant differences (*p* < .05), *n* = 3. “MM”: Minced meat; “HM”: Fish head meat; “R”: Fish roe; “1:4”: Under feed liquid ratio1:4; “A”: Under feed liquid ratio1:2; “B”: Under feed liquid ratio1:2, using A as the extract liquid; “C”: Under feed liquid ratio1:2, using B as the extract liquid; and “D”: Under feed liquid ratio1:2, using C as the extract liquid

The presence of taste amino acids and taste nucleotides produce a synergistic effect and can significantly improve the umami taste. The equivalent umami concentration (EUC, g MSG/100 g) is the concentration of MSG equivalent to the umami intensity given by the mixture of MSG‐like amino acids. According to the EUC in Figure 3, all samples significantly increased with repeated heat extraction (*p* < .05). And MM samples in the “D” condition, HM and R samples in “C” condition, reached the highest EUCs, respectively, were 1.37 g MSG/100 g, 0.87 g MSG/100 g and 0.49 g MSG/100 g. This was mainly because during the continuous heat extraction process, the content of IMP, AMP, Ala, and Glu all significantly increased, especially in the last thermal extraction (Tables 3 and 4), the soluble proteins in the sample such as myosin, sarcoplasmic protein, and actin were continuously dissolved, and these dissolved proteins were degraded to produce amino acids, peptides, and the others. With the accumulation of times, the water‐soluble taste substance in the extract was getting richer and thicker, especially in the last concentration process (Tang et al., 2015). It also showed that the “D” condition of MM samples have better umami taste than other state of the water‐soluble taste substance. The taste threshold of MSG is 0.03 g/100 g; thus, all samples have greater umami intensity than it. The EUCs in the “D” state of MM samples were 1.37 g MSG/100 g, it meant equivalent to umami intensity produced by 1.37 g MSG in 100 g water‐soluble taste substance. At this time, the synergistic action produced a very strong umami taste in the water‐soluble taste substance.

## 4 CONCLUSION

This study demonstrated that the umami amino acid content of the water‐soluble taste substance could reach the original sample. The umami amino acid content of the water‐soluble taste substance in the “D” condition extracted from the minced meat was equivalent to the original sample content. However, the fish head meat and the fish roe were in the “C” state. In addition, the total content of umami amino acids and IMP all gradually increased during the concentration process. Besides, the “D” condition of MM samples, “C” state of HM samples, and R samples were 1.37 g MSG/100 g, 0.87 g MSG/100 g, and 0.49 g MSG/100 g, respectively, which reached the highest EUCs. Therefore, we could make full use of these water‐soluble substances; for example, adding them to freshwater surimi and then surimi gel products with seawater fish taste characteristics.

## CONFLICT OF INTEREST

The authors declare that they have no conflict of interest.

## ETHICAL STATEMENT

The authors state that human and vertebrate animal testing was unnecessary in this study.
